# Predicting the submission frequency of periodic safety update reports: development and application of the EURD tool

**DOI:** 10.3389/fmed.2024.1299190

**Published:** 2024-02-08

**Authors:** María Gordillo-Marañón, Gianmario Candore, Marta López-Fauqued, Katerina-Christina Deli, Loris Piccolo, Paolo Alcini, Tom Paternoster-Howe, Irene Rager, Robin Ruepp, Menno van der Elst

**Affiliations:** ^1^Healthcare Data Workstream, Data Analytics and Methods Task Force, European Medicines Agency, Amsterdam, Netherlands; ^2^Institute of Cardiovascular Science, Faculty of Population Health, University College London, London, United Kingdom; ^3^Vaccines and Therapies for Infectious Diseases Office, Human Medicines Division, European Medicines Agency, Amsterdam, Netherlands; ^4^Referrals Office, Human Medicines Division, European Medicines Agency, Amsterdam, Netherlands; ^5^Dutch Medicines Evaluation Board, Utrecht, Netherlands

**Keywords:** pharmacovigilance, drug safety, periodic safety update report, EURD list, data driven, prediction

## Abstract

**Introduction:**

Periodic Safety Update Reports (PSURs) are a key pharmacovigilance tool for the continuous evaluation of the benefit–risk balance of a medicinal product in the post-authorisation phase. The PSUR submission frequency for authorised active substances and combinations of active substances across the EU is individually determined. The objective of this research was the development and application of the EURD tool, a statistical method based on readily available safety data to predict PSUR frequencies and to ensure a consistent risk-based approach.

**Methods:**

First, variables considered relevant in determining the PSUR frequency were identified from data sources available at the European Medicines Agency. A subsequent first survey with National Competent Authorities in Europe lead to a prioritisation of identified variables, while a second survey was carried out to propose the PSUR frequencies for a set of substances. Finally, a regression model was built on the information collected, applied to a larger list of substances and its results tested via a third survey with the same experts.

**Results:**

The developed EURD tool was applied to the 1,032 EURD list entries with a PSUR assessment deferred to 2025 at the time of the creation of the list in 2012. As the number of procedures would have had a significant impact on the workload for the European Medicines Regulatory Network (EMRN), in a second step the workload impact was estimated after allocating the entries according to their proposed frequency. The analysis suggests that all entries could be reviewed by 2038 by increasing the median workload by 15% (from 868 to 1,000 substances/year).

**Conclusion:**

The EURD tool is the first data-driven application for supporting decision making of PSUR frequencies based on relevant active substance safety data. While we illustrated its potential for improving the assignment of PSUR submission frequencies for active substances authorised in the EU, other institutions requiring periodic assessment of safety data and balancing of the resulting workload could benefit from it.

## 1 Introduction

Periodic Safety Update Reports (PSURs) are legally required pharmacovigilance documents intended to provide an evaluation of the benefit–risk balance of a medicinal product at defined time points after its authorisation (1, 2).

The objective of a PSUR is to present a comprehensive, concise and critical analysis of the benefit–risk balance of the medicinal product, taking into account new or emerging information in the context of cumulative information on risk and benefits.

Before 2012, PSUR submission frequencies followed a standardised scheme (every 6 months for the first 2 years of market experience, then annually for the following 2 years, followed by 3-yearly submissions).

In 2012, as a consequence of the EU Pharmacovigilance legislation, the European Medicines Agency (EMA) created and published the first version of the list of European Union reference dates (EURD list) which included the submission frequency of PSURs for authorised active substances and combinations of active substances across the EU (3, 4). The creation of the EURD list aimed at harmonising data lock points (DLPs, cut-off date for data to be included in a PSUR) and frequencies of submission of PSURs for the same active substance and combination of active substances, to allow a single EU assessment of the risk–benefit balance of an active substance based on all available data.

Marketing Authorisation Holders for products containing active substances and combinations of active substances, referred to hereafter as EURD list entries or simply “substances”, that are subject to a PSUR single assessment (PSUSA) at European Union (EU)/ European Economic Area (EEA) level must submit the relevant PSURs according to the requirements set up in “EURD list” (5–7). The European Union Reference Date is the date of the first marketing authorisation in the EU of a medicinal product containing that substance; or if the date of first marketing authorisation cannot be ascertained, the earliest of the known dates of the marketing authorisations in the EU for a medicinal product containing that substance.

The frequency for submission of PSURs is based on the risk-based approach defined in the Guideline on good pharmacovigilance practices Module VII (8), and considers the following prioritisation criteria:

- information on risks or benefits that may have an impact on the public health;
- new products for which there is limited safety information available to date (includes pre- and post-authorisation experiences) and/or subjected to additional monitoring;
- significant changes to the product (e.g., new indication has been authorised, new pharmaceutical form or route of administration broadening the exposed patient population);
- vulnerable patient populations/poorly studied patient populations, missing information not available at the time of authorisation (e.g., children, pregnant women) while these populations are likely to be exposed in the post-authorisation setting;
- signal of/potential for misuse, medication error, risk of overdose or dependency;
- the size of the safety database and exposure to the medicinal product.

Any change in these criteria for a given substance may lead to an amendment of the respective entries in the EURD list (e.g., increase or decrease of the frequency for PSUR submissions).

At the time of the EURD list’s creation in 2012, the majority of substances authorised in more than one Member State were allocated frequencies up to 12 years based on the criteria mentioned above. However, 1,880 out of a total of 3,349 entries, consisting mostly of long-time authorised products without pertinent safety issues requiring frequent assessment, were allocated a PSUR submission frequency of 13 years with a DLP in 2025. Since its creation, the EURD list has been monthly updated, including the deletion of substances that are no longer authorised, the merging or splitting of entries and the addition of new substances. As of 2 February 2022, the EURD list governed PSUR submission frequencies for 3,085 active substances and combinations across the EU. Of these, 1,188 entries remain with a PSUR submission frequency of 13 years and a DLP of 2025. The submission of such a high volume of entries in a single year would pose challenges to the EMA and the European Medicines Regulatory Network (EMRN). Consequently, a risk-based scientifically sound redistribution of the entries over time will prevent a workload peak while maintaining an appropriate assessment frequency for each substance.

To support the assignment of PSUR frequencies of the 1,188 EURD list entries and to ensure a consistent approach across entries, the EU Pharmacovigilance Risk Assessment Committee (PRAC) called for the development of a statistical tool (the EURD tool) with the evaluation criteria based on readily available safety data (9). Such safety data would be sourced from the European database of suspected adverse drug reaction reports (EudraVigilance, EV), from the EURD list (10), from the European pharmacovigilance issues tracking tool (EPITT) (11), and from the EMA’s internal Formal Referrals European Union Database (FREUD) (12).

This article aims to describe the development and application of the EURD tool, a statistical method based on readily available safety-relevant data from the above databases to provide proposals for PSUR submission frequencies for a set of EURD list entries for which PSUR assessment was deferred to 2025. To estimate the impact on the workload of allocating those entries for EMA and the EMRN, we performed additionally a series of simulations under different statistical principles for the allocation of PSUR periodicity to substances. Lastly, we suggest how the EURD tool, or similar data-driven approaches, can be used to support other institutions requiring periodic assessment of safety data.

## 2 Materials and methods

### 2.1 Research design

The tool was initially developed to support in general risk-based decision making for PSUR frequencies in line with the requirements of GVP module. We describe here how it is currently being used in an exercise to review PSUR cycles for EURD list entries deferred to 2025 with previously allocated 13 year frequencies.

**Figure 1:**
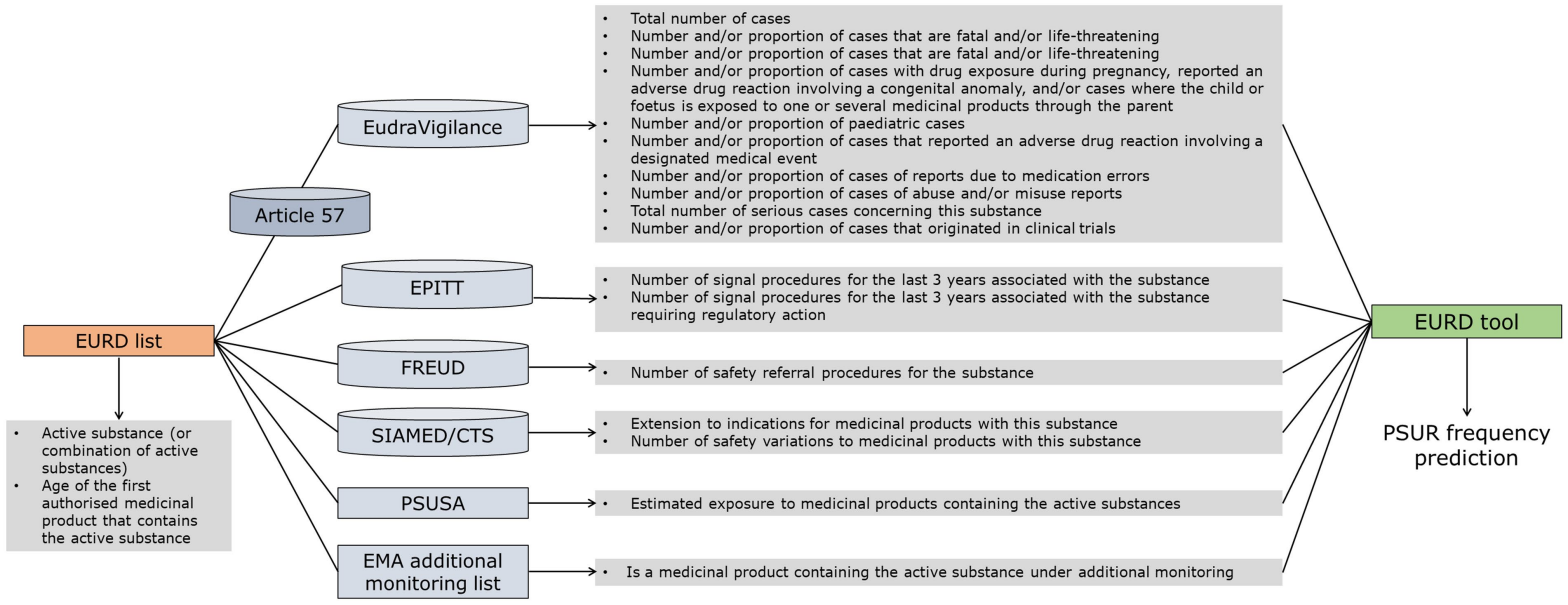
Relations between data sources and criteria feeding into the proposed methodology. SIAMED – EMA’s internal database for centrally authorised products, CTS – Communication and Tracking System, used by the NCAs for the licensing of human and veterinary medicinal products via the mutual recognition and decentralised procedures, PSUSA-PSUR single assessment procedure.

Data from different databases were considered and/or sourced when developing the EURD tool. Figure 1 illustrates the connection between the EURD list (starting point) and the type of variables present in each database. While all the variables were considered during the selection of the modelling strategy phase, data were only extracted for the variables considered relevant for the development of the model by representatives from EU/EEA national competent authorities (NCAs).

### 2.2 EURD list

As of 2 February 2022, the EURD list included 3,085 entries for active substances and combinations of active substances. Of these, 1,188 entries, mainly substances authorised for several decades, had a DLP of 2025 and a PSUR submission frequency of 13 years.

To allow for the extraction of safety-related data from EV (11, 13), the listed entries were mapped to the “Article 57 database” [EudraVigilance medicinal product dictionary (XEVMPD)], which contains information on all medicinal products and corresponding active substances authorised in the European Economic Area (EEA) (14), serving as the controlled vocabulary against which the terms reported in the safety reports, often as free text, are mapped. The extraction was performed using the “active substance high level” criterion from the medicinal product hierarchy, as this is the most aligned in terms of granularity (e.g., grouping of the various salts of a substance) with the EURD list.

Out of the 1,188 deferred entries, 67 could not be mapped to the Article 57 database, thus their PSUR frequencies will need to be individually assessed through Granularity and Periodicity Advisory Group (GPAG) consultation, a dedicated working group that supports PRAC (15), rather than through the tool. On the other hand, eleven non-deferred entries were mapped to the same ‘active substance high level’ as some of the entries deferred to 2025 and were included in the analysis. This is because the ‘active substance high level’ criterion could include multiple active substances (13). Therefore, the final dataset analysed in this report included 3,085 substances in the EURD list, of which 1,132 were deferred to 2025 requiring PSUR frequency proposals.

### 2.3 Selection of the modelling strategy

The criteria for the model selection were based on results from two surveys with EU/EEA NCAs. In the first survey, the EURD contact points across EU/EEA Member States were requested to score certain criteria that could be derived from databases hosted by EMA based on their relevance for decision making of PSUR frequencies (Table 1). Eight out of the 17 variables were considered relevant for the development of the model, based on the response of 24 representatives from 16 NCAs (Table 2).

**Table 1:** Criteria included in the first survey to the EURD contact points across EU/EEA Member States.

Criteria	Data source
Number of signal procedures for the last 3 years associated with the substance	EPITT (11)
Number of signal procedures for the last 3 years associated with the substance requiring regulatory action	EPITT (11)
Age of the first authorised medicinal product that contains the active substance	EURD list
Estimated exposure to medicinal products containing the active substances	Last PSUSA
Number of safety referral procedures for the substance	FREUD (12)
Extension to indications for medicinal products with this substance	EMA’s product information and application tracking system [SIAMED (16)]. Data readily available only for centrally authorised products
Number of safety variations to medicinal products with this substance	EMA’s product information and application tracking system [SIAMED (16)]. Data readily available only for centrally authorised products
Is a medicinal product containing the active substance under additional monitoring	EMA additional monitoring list (17)
Total number of cases	EudraVigilance (11)
Number and/or proportion of cases that are fatal and/or life-threatening	EudraVigilance (11)
Number and/or proportion of cases with drug exposure during pregnancy, reported an adverse drug reaction involving a congenital anomaly, and/or cases where the child or foetus is exposed to one or several medicinal products through the parent (parent–child)	EudraVigilance (11) (details in Supplementary Information 1)
Number and/or proportion of paediatric cases	EudraVigilance (11)
Number and/or proportion of cases that reported an adverse drug reaction involving a designated medical event (18)	EudraVigilance (11)
Number and/or proportion of cases of reports due to medication errors	EudraVigilance (11)
Number and/or proportion of cases of abuse and/or misuse reports	EudraVigilance (11)
Total number of serious cases concerning this substance	EudraVigilance (11)
Number and/or proportion of cases that originated in clinical trials	EudraVigilance (11)

**Table 2:** Correlation coefficients between the variables selected in the first survey and the PSUR frequencies proposed in the second survey.

Criteria	Pearson’s correlation coefficient
Age of the first authorised medicinal product that contains the active substance	0.48
Number of signal procedures for the last 3 years associated with the substance requiring regulatory action	−0.39
Number of safety referral procedures for the substance	−0.22
Total number of cases	−0.45
Number of cases that are fatal and/or life-threatening	−0.41
Number of cases with drug exposure during pregnancy, reported an adverse drug reaction involving a congenital anomaly, and/or cases where the child or foetus is exposed to one or several medicinal products through the parent (parent–child)	−0.19
Number of paediatric cases	−0.26
Number of cases that reported an adverse drug reaction involving a designated medical event (18)	−0.37

**Figure 2:**
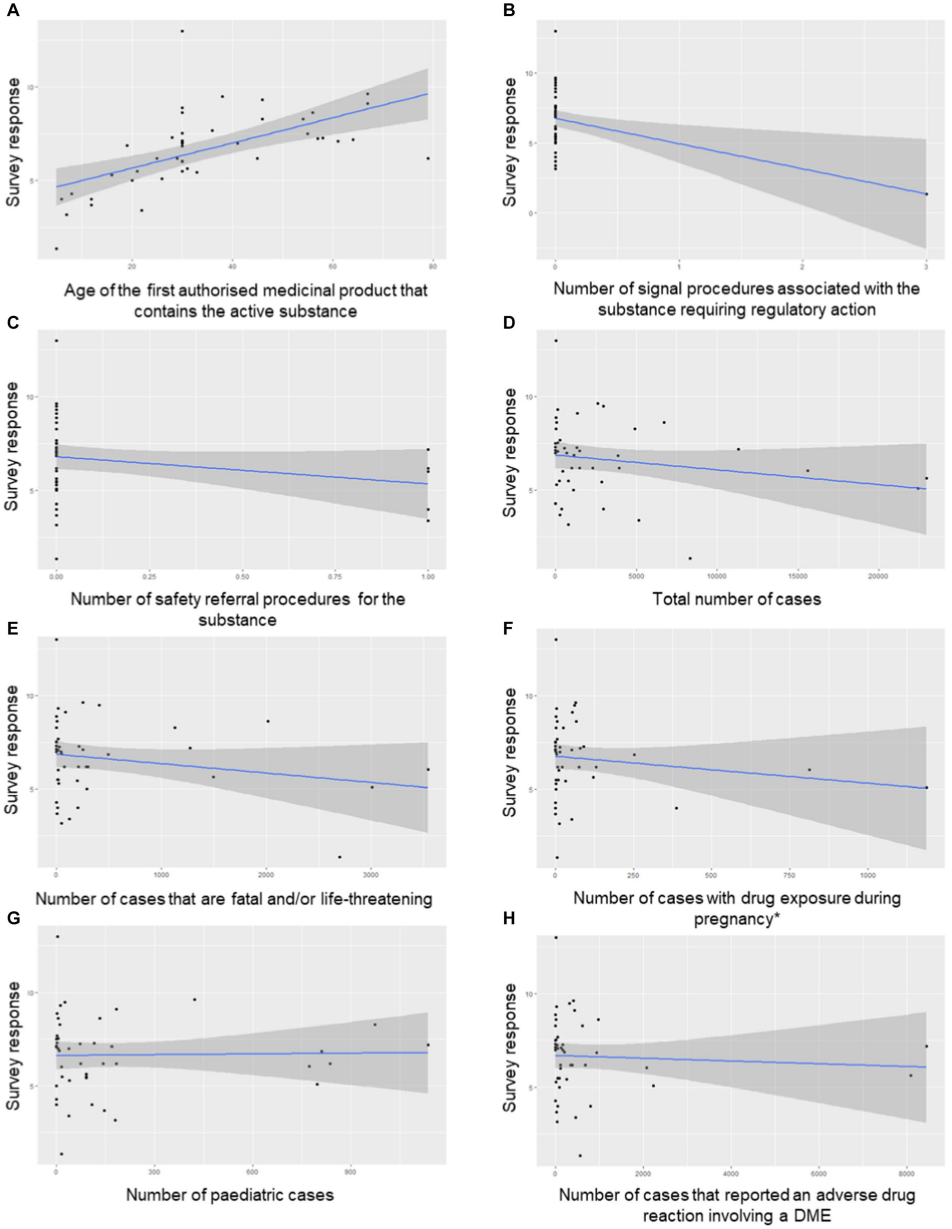
Correlation plots **(A-H)** of the variables investigated and the PSUR frequencies proposed for the 45 EURD list entries in the test dataset. The asterisk (*) in subfigure **F** indicates number of cases with drug exposure during pregnancy, reported an adverse drug reaction involving a congenital anomaly, and/or cases where the child or foetus is exposed to one or several medicinal products through the parent (parent–child). DME, designated medical event.

In a second survey, the same panel of representatives were asked to propose PSUR frequencies for 45 substances or combinations listed in the EURD list that were selected to cover a wide range of therapeutic areas including nationally and centrally authorised medicinal products. An exploratory work was then performed to investigate the correlation between the geometric means of the proposed PSUR frequencies and the selected criteria from the 1st survey using the Pearson method. The geometric means were selected since the data were right-skewed. Figure 2 shows the correlation plots between the variables investigated and the PSUR frequencies proposed, with the Pearson’s correlation coefficients detailed in Table 2.

Of note, the variable ‘Estimated exposure to medicinal products containing the active substances (if available)’ was excluded from the correlation analysis as such information was only available for a limited set of entries which had undergone a previous PSUSA, and for those it may cover only in few cases generics.

### 2.4 Regression model

The selected criteria (see Table 2) were then used to develop a regression model. To avoid the inclusion of correlated variables that could lead to multicollinearity, we calculated the pair-wise correlation between the selected criteria. It was observed that the total number of cases from EV was highly correlated with the number of cases reported to EV for the last 3 years that are fatal and/or life-threatening (*r^2^* = 0.93) and the number and/or proportion of cases that reported an adverse drug reaction involving a designated medical event for the last 3 years (*r^2^* = 0.82) and thus, only the total number of cases reported to EV for the last 3 years was included in the model.

Similarly, the number and/or proportion of cases with drug exposure during pregnancy, reported an adverse drug reaction involving a congenital anomaly, and/or cases where the child or foetus is exposed to one or several medicinal products through the parent (parent–child) reported to EV for the last 3 years was highly correlated with the number and/or proportion of paediatric cases (*r^2^* = 0.89), and the latter was retained for the model.

Next, the data were transformed to natural logarithms to normalize skewness and a linear regression model was developed using a training dataset of 45 EURD entries.

The natural logarithm (1 + x) of the PSUR submission frequency was modelled via the natural logarithm (1 + x) of years in the EURD list (*Years_EURD*), number of signal procedures that required regulatory action for the last 3 years (*Signals_regulatory_action*), number of safety referral procedures (*N_Safety_Referrals*), total number of cases reported to EV for the last 3 years (*Cases*) and cases of drug exposure during pregnancy reported to EV for the last 3 years *(Pregnancy)*, as follows:

$\begin{array}{l} \mathrm{lm}(\log1p\;\left(\mathrm{Survey}\_\mathrm{average}\right)\sim \log1p\;\left(\mathrm{Years}\_\mathrm{EURD}\right)\\ +\log1p\;\left(\mathrm{Signals}\_\mathrm{regulatory}\_\mathrm{action}\right)\\ +\log1p\;\left(N\_\mathrm{Safety}\_\mathrm{Referrals}\right)+\log1p\;\left(\mathrm{Cases}\right)\\ +\log1p\;\left(\mathrm{Pregnancy}\right)) \end{array}$

where *log1p* was used to return the natural logarithm of one plus the input array for each of the variables to avoid errors in the model due to zero values in certain variables.

The adjusted R square was used to estimate the goodness-of-fit of the model. The linearity assumption was inspected via the residual plot (a scatter plot of the residuals against the fitted values) and the homoscedasticity (i.e., the constant variance of errors or residuals across the range of predictor variables) tested using the Breusch-Pagan test (19).

The model using the above variables (listed also in Table 3) was applied on a set of a further 118 EURD list entries and the output reviewed through a subsequent survey to EURD contact points across the EU/EEA Member States.

**Table 3:** Associations between variables and proposed PSUR frequency in the training dataset after transforming the estimates back to the natural value.

Variable	Estimate (95% CI)	*p*-value
(Intercept)	0.99	–
Age of the first authorised medicinal product that contains the active substance	0.41 (0.28; 0.55)	<0.001
Number of signal procedures for the last 3 years associated with the substance requiring regulatory action	−0.34 (−0.51; −0.11)	0.007
Number of safety referral procedures for the substance	−0.13 (−0.32; 0.12)	0.263
Total number of cases reported to EV for the last 3 years	−0.02 (−0.05; 0.025)	0.454
Number of cases with drug exposure during pregnancy, reported an adverse drug reaction involving a congenital anomaly, and/or cases where the child or foetus is exposed to one or several medicinal products through the parent (parent–child) which were reported to EV for the last 3 years	−0.02 (−0.07; 0.03)	0.385

### 2.5 Workload simulations

Three simulations were performed under two different scenarios to estimate the additional workload generated for EMA and the EMRN by allocation of the deferred 1,132 EURD list entries for PSUR assessment. The median number of PSUR submission for the time period 2022–2032 was estimated in 868 entries per year, based on the predicted submissions of the EURD list entries excluding those deferred to 2025.

In scenario 1, the PSUR frequencies for substances or combinations of active substances not included in the set of 1,132 entries deferred to 2025 remained unchanged. In scenario 2, the PSUR frequencies for the substances with a cycle of 2, 3 or 4 years was extended to 5 years. An extension of the PSUR cycle was not supported by GPAG for the EURD list entries with a PSUR frequency of a year or less due to the close safety monitoring needed, particularly for newly authorised products with new active substances, which are assigned a 6-monthly cycle.

The following simulations were performed in each scenario and were defined based on the following rationale:

1. Allocating the substances from 2022. As the minimum predicted frequency is 5 years, the first substances would be reviewed in 2027.
2. Allocating up to 1,000 substances per year, starting in 2022. The DLP for substances would be set in 2022 based on available capacity.
3. Allocating up to 955 substances per year, starting in 2022. This number represents approximately a 7.5% increase of the estimated median number of submissions per year (e.g., 868 entries per year) and was considered as a compromise between simulation 2 and the estimated current median workload. The DLP for substances would be set in 2022 based on available capacity.

**Figure 3:**
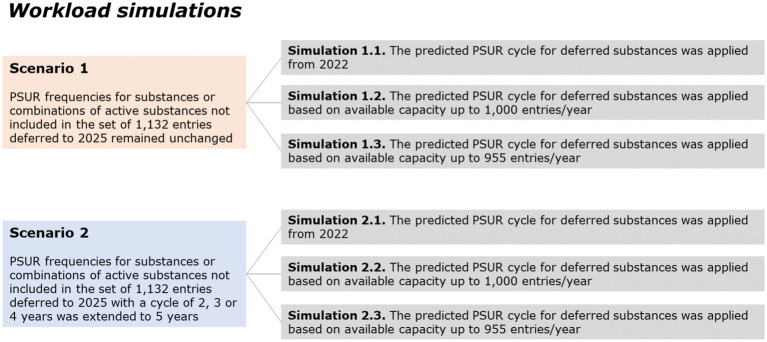
Scenarios used for workload simulations.

The workload simulations did not take into account the standard updates to the EURD list (e.g., newly authorised products, removal of substances for example, when products containing them are no longer authorised in EU, or merging of existing entries). Thus, it was taken as an assumption that introducing new substances per year does not substantially impact the total workload. Figure 3 illustrates how the workload simulations were performed.

## 3 Results

### 3.1 Development and validation of the regression model

The coefficients and *p*-values for each of the variables in the regression model are shown in Table 3. Despite the few observations used to develop the model (*n* = 45), the results indicated that the predicted frequency is largely driven by the age of the first authorised medicinal product that contains the active substance, with every coefficient for the other variables in the expected direction of effect. The largest effect was observed for the number of signal procedures for the last 3 years requiring regulatory action, as the presence of one signal procedure requiring regulatory action reduced the predicted PSUR frequency by approximately 5 months (0.41 years). On the other hand, approximately 21 cases from spontaneous reports were needed to obtain a similar a reduction [i.e., 5 months/(0.02 * 12 months)].

The adjusted R square obtained in the model indicated that up to 70% of the variance in the PSUR submission frequency could be explained by the variables included in the model. The residual plot indicated a linear relationship and the significance of the Breusch-Pagan test (*p*-value = 0.30) suggested homoscedasticity, therefore, the assumptions of the linear regression held true.

The model was applied on a set of a further 118 EURD list entries and the output reviewed through a subsequent survey to EURD contact points across the EU/EEA Member States, of whom 16 responded and confirmed unanimously the proposed PSUR frequencies for 79 EURD list entries. For the remaining 39 entries, 15 EU Member States agreed with the proposed frequencies for 27 entries, while 14 EU Member States agreed with the proposed frequencies for the other 12 entries. Since most of the EU Member States agreed with the proposals by the EURD tool, the model was endorsed by PRAC.

**Figure 4:**
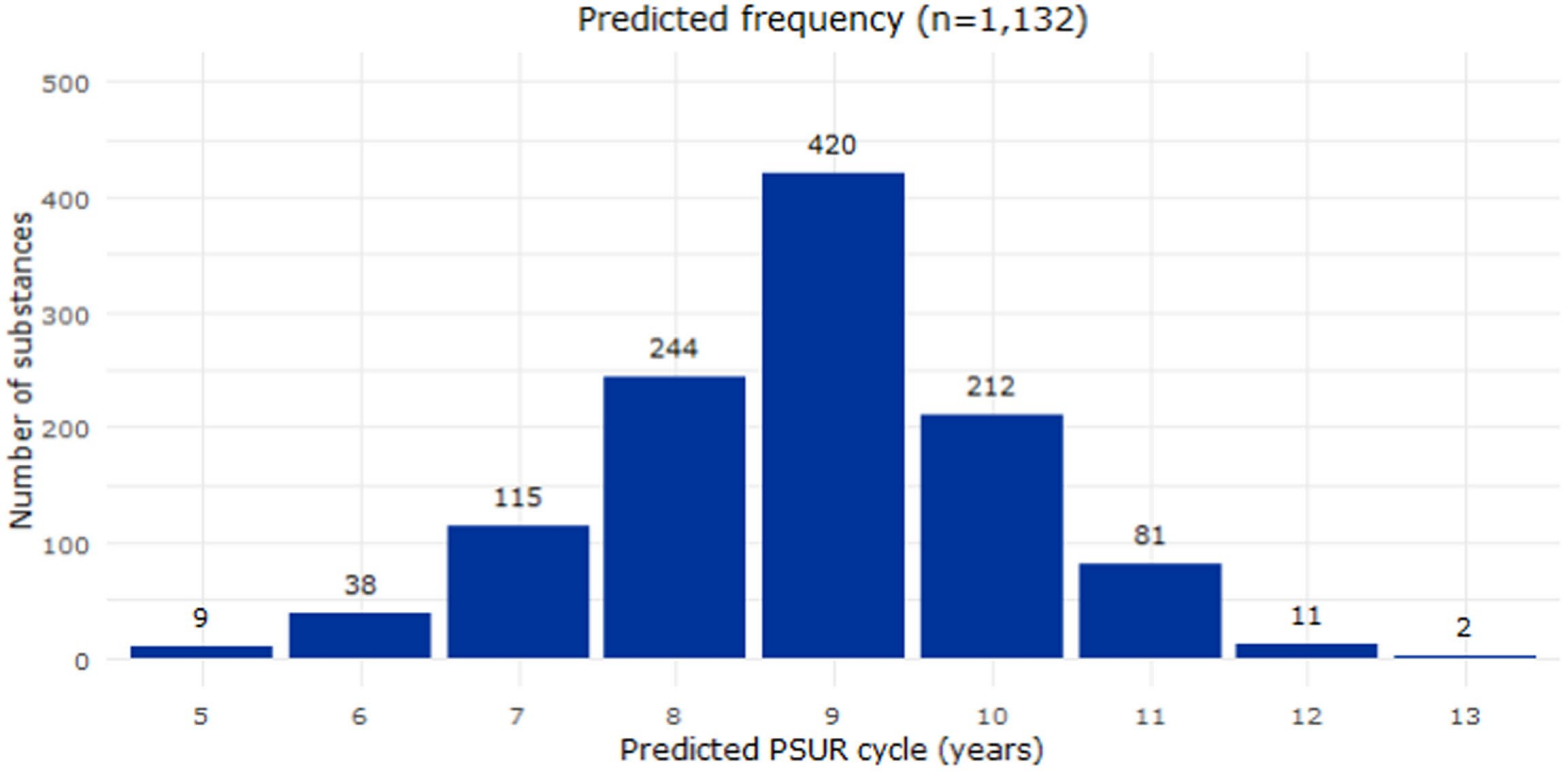
Distribution of the predicted frequency submission of the 1,132 deferred substances (EURD list entries). PSUR, periodic safety update report.

**Figure 5:**
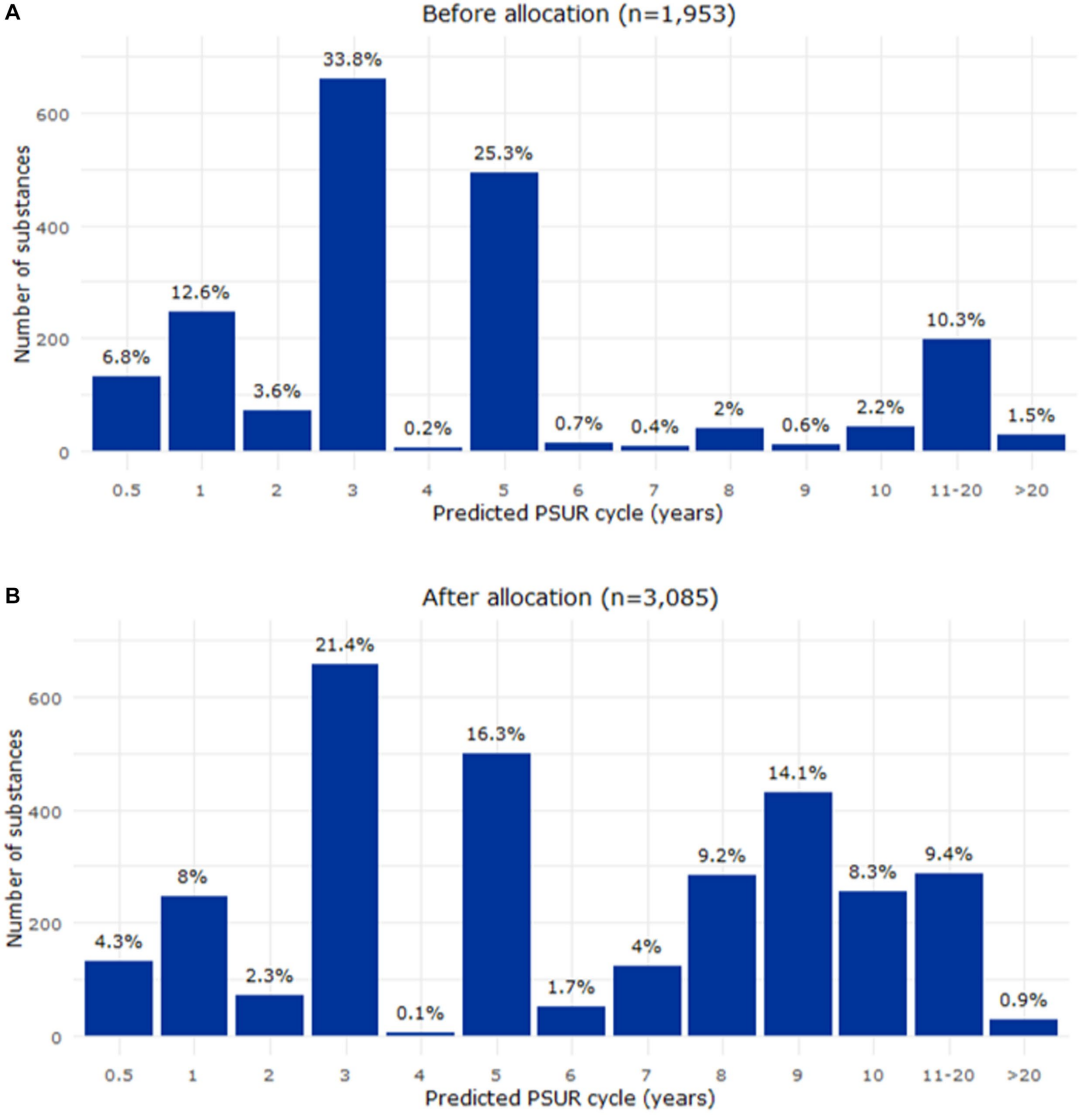
Relative distribution of the PSUR frequencies before **(A)** and after **(B)** the allocation of the deferred substances (EURD list entries). PSUR, periodic safety update report.

After endorsement, the model was applied to the EURD list entries deferred to 2025 to predict PSUR submission frequencies based on their safety profile (Figure 4). The final dataset analysed in this report included 3,085 substances in the EURD list, of which 1,132 were deferred to 2025 requiring PSUR frequency proposals. The model predicted for the majority of the substances (77.4%) a PSUR cycle between 9 and 10 years. After allocating the impacted substances to the overall distribution of PSUR frequencies across the EURD list, an increased proportion of the substances had a longer PSUR cycle than before the allocation (Figure 5). For example, before allocation of the deferred substances, 17% of the EURD list entries had a PSUR frequency of 7 years or longer, which increased to 46% after allocation of the deferred substances.

### 3.2 Evaluation of the impact on the EMRN through workload simulations

A series of simulations were performed to evaluate the impact on the workload of the EMRN after allocating the substances deferred to 2025. Three simulations were conducted under two different scenarios as detailed in Methods.

#### 3.2.1 Scenario 1

**Figure 6:**
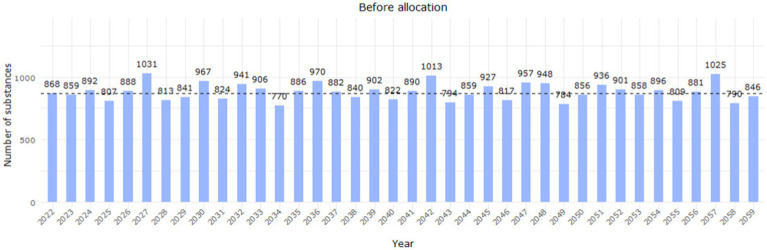
Workload distribution per year before the allocation of deferred substances (EURD list entries) up to 2059. The dashed line indicates the estimated median workload between 2022 and 2032 (868 entries/year based on the data lock point of the entry in the EURD list).

Under this scenario, the baseline median number of PSUR submissions for the time period 2022–2032 was 868 entries per year (Figure 6).

**Figure 7:**
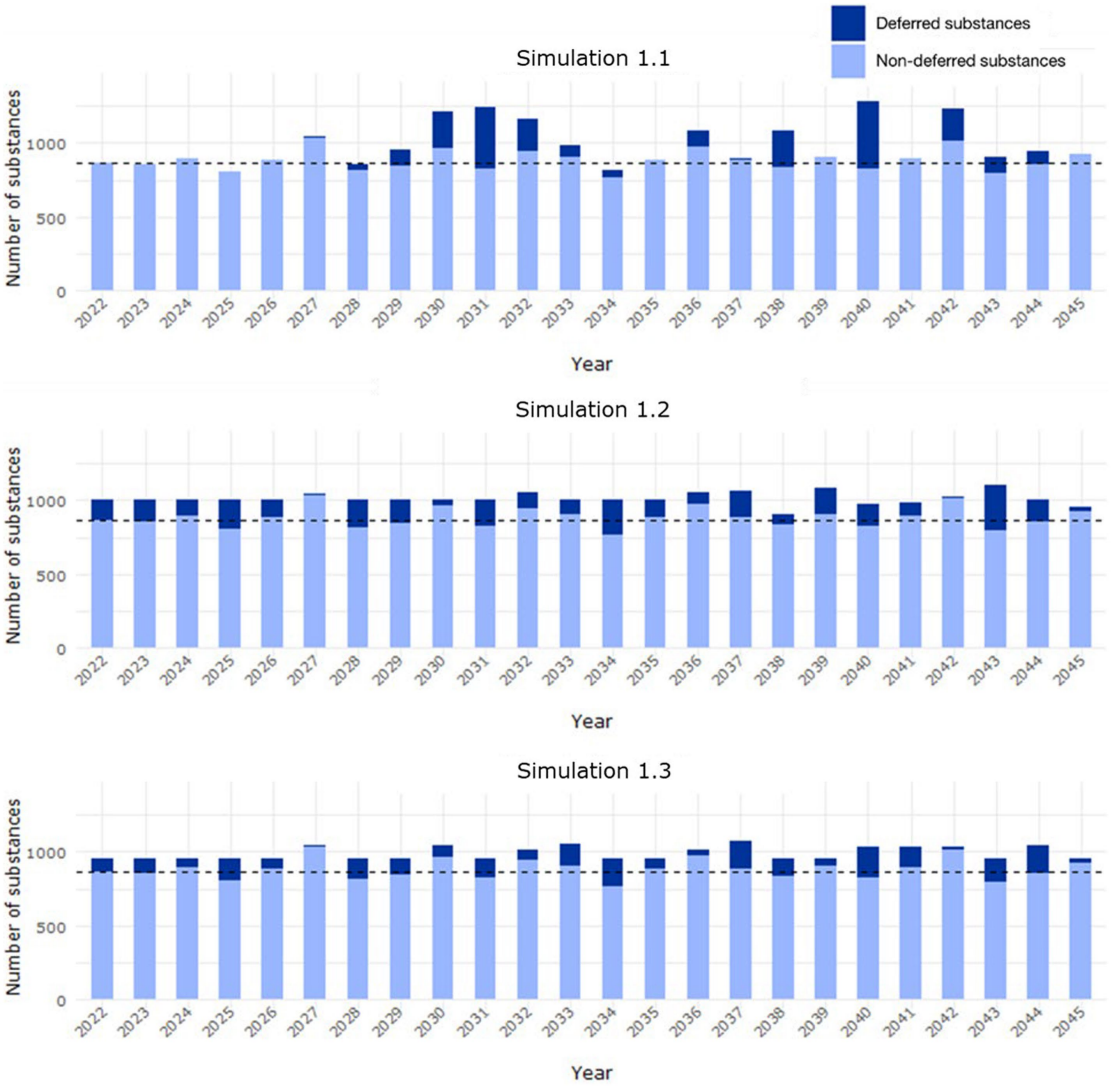
Workload distribution in Scenario 1 from 2022 to 2045, where the predicted PSUR cycle for deferred substances (EURD list entries) was applied (i) from 2022 (simulation 1.1), (ii) based on available capacity up to 1,000 entries/year (simulation 1.2), (iii) available capacity up to 955 entries/year (simulation 1.3). The median background workload of 868 entries/year is indicated with the dashed line (based on the data lock point of the entry in the EURD list).

The first simulation (simulation 1.1) evaluated the application of the predicted PSUR cycle for the deferred entries from 2022. This would imply that, for instance, an entry with a predicted cycle of 9 years would be first reviewed in 2031. Under this condition, all the entries would have been reviewed by 2035. Regarding workload, there would be between 100–300 (approximately 12%–35% of the estimated median workload) more substances to assess per year in the years 2029–2032 (Figure 7, simulation 1.1).

The second simulation (simulation 1.2) evaluated the application of the predicted PSUR cycle, starting from 2022, based on available capacity. If the maximum number of submissions per year is set to the current median number of submissions per year (i.e., 868 EURD list entries/year), by 2,100 some substances would still not have been reviewed. Therefore, a target of up to 1,000 submissions per year (approximately a 15% increase of the estimated median workload) was considered. Under this condition, all entries would have been reviewed by 2038 (Supplementary Table S1). An even workload distribution was observed under this condition (Figure 7, simulation 1.2), with an increase during some years of up to 1,100 entries/year (+10% further increase; 26% of the estimated median workload), which is above the threshold of 1,000 entries due to the cumulative effect of subsequent PSUR submissions once the entries have been initially allocated.

In the third simulation (simulation 1.3), the new PSUR cycle was applied based on capacity up to a target of 955 submissions per year. Under this condition, all the EURD list entries would have been reviewed by 2059 (Supplementary Table S2). Regarding the workload, although an even distribution is observed, in some years the number of PSUR submitted would further increase by up to 20%, thus exceeding 1,000 entries/year (Figure 7, simulation 1.3).

#### 3.2.2 Scenario 2

**Figure 8:**
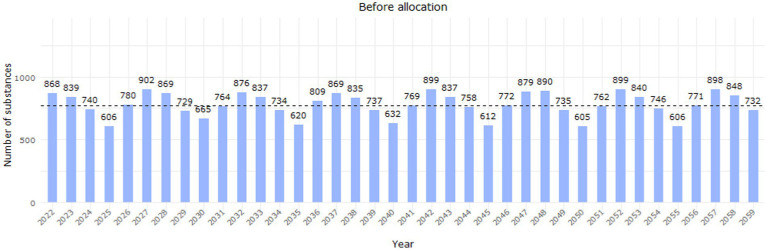
Workload distribution before the allocation of deferred substances (EURD list entries) up to 2059, after extending the cycle to 5 years for non-deferred entries with a PSUR frequency of 2, 3 or 4 years. The yearly distribution is based on the data lock point of the entries in the EURD list.

The extension of the cycle under this scenario impacted 733 substances (35% of all substances) and decreased the median workload per year to 772 PSUR assessments (Figure 8).

**Figure 9:**
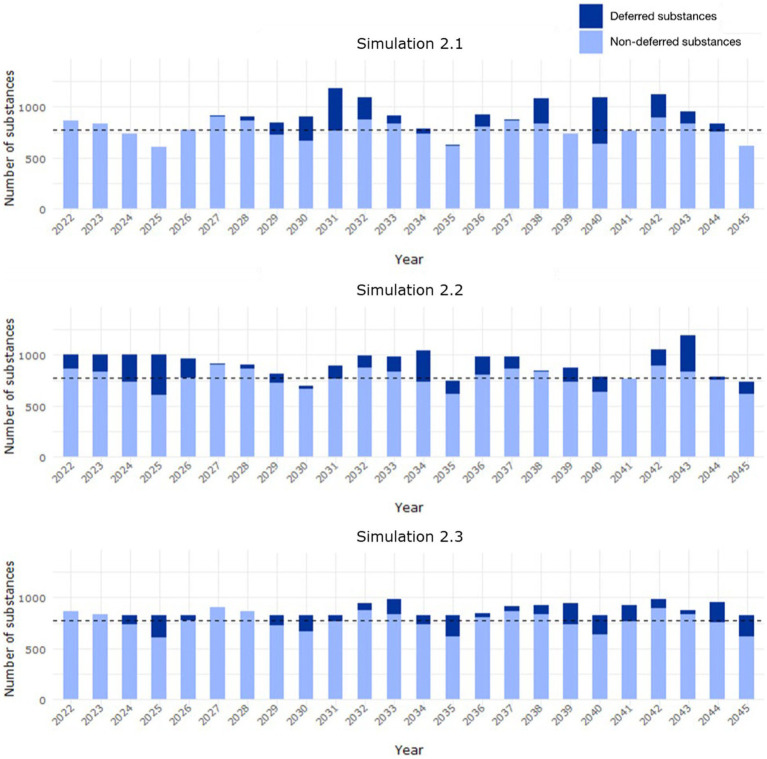
Workload distribution in Scenario 2 from 2022 to 2045, where the predicted PSUR cycle for deferred substances (EURD list entries) was applied (i) from 2022 (simulation 2.1), (ii) based on available capacity up to 1,000 entries/year (simulation 2.2), (iii) available capacity up to 955 entries/year (simulation 2.3). The median background workload of 772 entries/year is indicated with the dashed line. The yearly distribution is based on the data lock point of the entries in the EURD list.

In simulation 2.1, the predicted PSUR cycle was applied from 2022. Under this condition, all the entries would have been reviewed by 2035, and there would be between 100–300 more substances to review (approximately 14%–41% of the estimated median workload) between 2030–2033 (Figure 9, simulation 2.1).

Simulation 2.2 evaluated the application of the predicted PSUR cycle based on available capacity, up to a target of 1,000 submissions per year. Under this condition, all the entries would have been reviewed by 2026 (Supplementary Table S3). The workload is not as uniform as in simulation 1.2, and outliers were observed for certain years with over 1,000 or less than 700 substances to be assessed (Figure 9, simulation 2.2).

The last simulation (simulation 2.3) evaluated the application of the predicted PSUR cycle based on available capacity up to a target of 830 submissions per year. Under this condition, all the substances would have been reviewed by 2045 (Supplementary Table S4). Fluctuations in the workload were again observed (Figure 9, simulation 2.3), but with a smaller range than in simulation 2.3.

## 4 Discussion

The principles of risk-based decision making for PSUR frequencies are established and assigned through scientific assessments by EU pharmacovigilance experts based on the specified safety criteria detailed in the GVP Module VII (8). By integrating safety data from disparate sources, we developed a tool to predict PSUR submission frequencies and support decision making for determining PSUR frequencies of EURD list entries.

We illustrated the potential of the EURD tool by proposing the PSUR frequency for one third of the entries in the EURD list for which a submission frequency of 13 years and a DLP in 2025 was assigned at the time of the creation of the list. As of 2 February 2022, when the analysis was initiated, the EURD list governed PSUR submission frequencies for 3,085 substances across the EU. Of these, 1,188 were deferred to a DLP in 2025, of which 1,132 were in scope of this exercise. In addition to defining PSUR frequencies for these entries, we evaluated several scenarios to estimate the impact of allocating the deferred entries on the workload for EMA and the EMRN.

After applying the model to the entries deferred to 2025, 77.4% showed a predicted PSUR cycle between 9 and 10 years. Six different simulations were performed to estimate the increased workload per year. If the maximum number of submissions per year is set as the current median number of submissions (868 entries/year), by 2,100 some substances would have still not been reviewed. It was shown that by increasing the median workload by 15% (to an arbitrary level of 1,000 substances/year), all substances could be reviewed by 2038. A compromise could be reached by increasing the workload by 7.5%, which would allow the review of up to 955 substances per year and all substances would have been reviewed by 2059.

The tool has proven instrumental in redistributing the EURD list entries with a submission frequency of 13 years and a DLP in 2025 based on the PSUR frequency proposed by the model (20). The PSUR frequency and data lock points for these entries will be progressively updated based on the workload simulation 1.2, by increasing the median workload by 15%.

The EURD tool and the presented results are subject to some limitations. One of the criteria to identify the variables that could be included in the model was the readiness of the data. There could be other variables, for example the scope of the signal associated with the substance, that could influence the selection of a PSUR frequency in an individual assessment but were considered out of scope because of the difficulty to categorise the variable to allow its inclusion in the model. Nevertheless, the criteria initially proposed was reviewed and evaluated by representatives from the EU Members States and endorsed by the PRAC, indicating that such criteria were adequate to be included in the model and advise PSUR frequencies. In addition, the model was developed using a training dataset of only 45 substances, which affected the power to detect significant associations. However, while the age of the first authorised medicinal product that contains the active substance and the number of safety signal procedures for the last 3 years requiring regulatory action were the only variables significantly associated with the proposed PSUR frequency, the estimates for the other variables were in the expected direction of effect. Ultimately, the aim of the tool was to support decision-making, as a guide for driving transparency and harmonisation, and it was not developed as a prescriptive tool. In addition, the output of the simulations should be interpreted with some caution given the dynamic nature of the EURD list, which is updated monthly, and the workload simulations were based on entries in the EURD list as of 2 February 2022.

With this tool, we believe that a feasible workload for the EU/EEA network in terms of PSUSA assessment has been achieved, avoiding a large peak of PSUR assessments in 2025 and allowing sufficient capacity left for the EU network to carry out other assessments. We are not aware whether a similar statistical approach has been taken outside of EU/EEA. However, some regulators outside of EU/EEA usually align the PSUR reporting requirements and timeframes with those required by EMA. Consequently, an impact in countries outside of the EU/EEA cannot be excluded. We consider that the impact of this tool for patients is minimal as there is a robust EU pharmacovigilance system in place to monitor the benefits and risks of all authorised medicinal products, thereby ensuring their safe and effective use.

## 5 Conclusion

The EURD tool is the first data-driven application for defining PSUR frequencies based on relevant substance safety data. The predicted PSUR cycle is thus a composite indicator of the overall safety during the post-authorisation phase. We confirmed the utility of the prediction of the tool through a subsequent validation step. In November 2022, the PRAC endorsed the GPAG recommendations on the proposal for implementing in the EURD list the new PSUR frequencies predicted by the EURD tool in the set of EURD list entries which PSURs were deferred to 2025 at the time of the EURD list creation (20). This was a one-off exercise to address the surge in workload combined with reconsideration of the PSUR frequencies, but in principle the tool could be considered for supporting frequency changes for other entries in the EURD list if requested by the PRAC, e.g., during PSUSA assessments or other regulatory procedures affecting the PSUR cycle. While we illustrated its potential for improving the assignment of PSUR submission frequencies for substances authorised in the EU, other institutions requiring periodic assessment of safety data and balancing of the resulting workload could benefit from it.

The approach used to develop the EURD tool could also be explored for other types of decision-making applications to leverage existing data related to safety across a range of active substances in authorised medicinal products.

## Data availability statement

The raw data supporting the conclusions of this article will be made available by the authors, without undue reservation.

## Author contributions

MG-M: Conceptualization, Data curation, Formal analysis, Investigation, Methodology, Visualization, Writing – original draft, Writing – review & editing. GC: Conceptualization, Data curation, Formal analysis, Investigation, Methodology, Validation, Writing – review & editing. ML-F: Writing – review & editing. K-CD: Data curation, Writing – review & editing. LP: Conceptualization, Data curation, Methodology, Writing – review & editing. PA: Supervision, Writing – review & editing, Resources. TP-H: Conceptualization, Data curation, Investigation, Methodology, Writing – review & editing. IR: Conceptualization, Resources, Supervision, Writing – review & editing. RR: Conceptualization, Data curation, Investigation, Methodology, Project administration, Supervision, Writing – review & editing. ME: Conceptualization, Methodology, Validation, Writing – review & editing.
