# Supplementary material for: Predicting the submission frequency of periodic safety update reports: development and application of the EURD tool
Source: Front Med (Lausanne). 2024 Feb 8;11:1299190. doi: 10.3389/fmed.2024.1299190 (PMC10882634; doi:10.3389/fmed.2024.1299190)
Supplement: Supplementary file 1 [file Data_Sheet_1.docx]

**Supplementary Information 1**

Conditions used to identify a cases of drug exposure during pregnancy in EudraVigilance. All the conditions below should be met.

- Age below 50 years old
- At least one of the following conditions:
  - Administration route is equal to/is in ‘Intra-amniotic’, ‘Intracervical’, ‘Transplacental’
  - Gestation Period is not null
  - Seriousness Congenital Anomaly is equal to/is in Yes
  - Reaction high level term (HLT) is equal to/is in ‘Exposures associated with pregnancy, delivery and lactation’ and reaction preferred term (PT) is not equal to/is not in ‘Maternal exposure during breast feeding’
  - SMQ (Standardised MedDRA query) Level 2 is equal to/is in Foetal disorders (SMQ) (Narrow)
- Reported drug indication is not equal to/is not for ‘Contraception’, ‘Post coital contraception’, ‘Oral contraception’, ‘Exposure via breast milk’, ‘Pregnancy test’, ‘Menorrhagia’, ‘Menstruation irregularity’, ‘Premenstrual syndrome’, ‘Hormone therapy’, ‘Ectopic pregnancy termination’, ‘Progestin replacement therapy’, ‘Lactation disorder’, ‘Intrauterine contraception’, ‘Injectable contraception’, ‘Polymorphic eruption of pregnancy’, ‘Post abortion haemorrhage’, ‘Postpartum disorder’, ‘Selective abortion’, ‘Abortion infected’, ‘Artificial menopause’, ‘Neonatal cardiac failure’, ‘Pregnancy after post coital contraception’, ‘Pregnancy of unknown location’, ‘Pregnancy test positive’, ‘transdermal contraception’.

# Supplementary Figures and Tables

**Supplementary Table 1.** Allocation of EURD list entries in workload simulation 1.2 based on up to 1,000 available spots /year. Note that entries before allocation include non-deferred entries; entries after allocation include those entries plus deferred entries allocated in the previous years. The year is based on the data lock point of the entries in the EURD list.

| Year | EURD list entries before allocation | EURD list entries after allocation | Available spots |
| --- | --- | --- | --- |
| 2022 | 868 | 868 | 132 |
| 2023 | 859 | 859 | 141 |
| 2024 | 892 | 892 | 108 |
| 2025 | 807 | 807 | 193 |
| 2026 | 888 | 888 | 112 |
| 2027 | 1031 | 1040 | - |
| 2028 | 813 | 851 | 149 |
| 2029 | 841 | 926 | 74 |
| 2030 | 967 | 997 | 3 |
| 2031 | 824 | 935 | 65 |
| 2032 | 941 | 1058 | - |
| 2033 | 906 | 931 | 69 |
| 2034 | 770 | 976 | 24 |
| 2035 | 886 | 998 | 2 |
| 2036 | 970 | 1061 | - |
| 2037 | 882 | 1071 | - |
| 2038 | 840 | 849 | 151 |

**Supplementary Table 2.** Allocation of EURD list entries in workload simulation 1.3 based on up to 955 available spots/year. Note that entries before allocation include non-deferred entries; entries after allocation include those entries plus deferred entries allocated in the previous years. The year is based on the data lock point of the entries in the EURD list.

| Year | EURD list entries before allocation | EURD list entries after allocation | Available spots |
| --- | --- | --- | --- |
| 2022 | 868 | 868 | 87 |
| 2023 | 859 | 859 | 96 |
| 2024 | 892 | 892 | 63 |
| 2025 | 807 | 807 | 148 |
| 2026 | 888 | 888 | 67 |
| 2027 | 1031 | 1040 | - |
| 2028 | 813 | 851 | 104 |
| 2029 | 841 | 881 | 74 |
| 2030 | 967 | 1042 | - |
| 2031 | 824 | 845 | 110 |
| 2032 | 941 | 1013 | - |
| 2033 | 906 | 1054 | - |
| 2034 | 770 | 820 | 135 |
| 2035 | 886 | 941 | 14 |
| 2036 | 970 | 1010 | - |
| 2037 | 882 | 1070 | - |
| 2038 | 840 | 914 | 41 |
| 2039 | 902 | 923 | 32 |
| 2040 | 822 | 1033 | - |
| 2041 | 890 | 1038 | - |
| 2042 | 1013 | 1034 | - |
| 2043 | 794 | 911 | 44 |
| 2044 | 859 | 1047 |  |
| 2045 | 927 | 941 | 14 |
| 2046 | 817 | 959 | - |
| 2047 | 957 | 1061 | - |
| 2048 | 948 | 1052 | - |
| 2049 | 784 | 1074 | - |
| 2050 | 856 | 908 | 47 |
| 2051 | 936 | 1011 | - |
| 2052 | 901 | 1025 | - |
| 2053 | 858 | 957 | - |
| 2054 | 896 | 954 | 1 |
| 2055 | 809 | 962 | - |
| 2056 | 881 | 1018 | - |
| 2057 | 1025 | 1222 | - |
| 2058 | 790 | 1066 | - |
| 2059 | 846 | 878 | 77 |

**Supplementary Table 3.** Allocation of EURD list entries in workload simulation 2.2 based on up to 1,000 available spots/year. Note that entries before allocation include non-deferred entries; entries after allocation include those entries plus deferred entries allocated in the previous years. The year is based on the data lock point of the entries in the EURD list.

| Year | EURD list entries before allocation | EURD list entries after allocation | Available spots |
| --- | --- | --- | --- |
| 2022 | 868 | 868 | 132 |
| 2023 | 839 | 839 | 161 |
| 2024 | 740 | 740 | 260 |
| 2025 | 606 | 606 | 394 |
| 2026 | 780 | 780 | 220 |

**Supplementary Table 4.** Allocation of EURD list entries in workload simulation 2.3 based on up to 830 available spots/year. Note that entries before allocation include non-deferred entries; entries after allocation include those entries plus deferred entries allocated in the previous years. The year is based on the data lock point of the entries in the EURD list.

| Year | EURD list entries before allocation | EURD list entries after allocation | Available spots |
| --- | --- | --- | --- |
| 2022 | 868 | 868 | - |
| 2023 | 839 | 839 | - |
| 2024 | 740 | 740 | 90 |
| 2025 | 606 | 606 | 224 |
| 2026 | 780 | 780 | 50 |
| 2027 | 902 | 902 | - |
| 2028 | 869 | 869 | - |
| 2029 | 729 | 738 | 92 |
| 2030 | 665 | 703 | 127 |
| 2031 | 764 | 807 | 23 |
| 2032 | 876 | 948 | - |
| 2033 | 837 | 989 | - |
| 2034 | 734 | 793 | 37 |
| 2035 | 620 | 620 | 210 |
| 2036 | 809 | 847 | - |
| 2037 | 869 | 911 | - |
| 2038 | 835 | 928 | - |
| 2039 | 737 | 945 | - |
| 2040 | 632 | 655 | 175 |
| 2041 | 769 | 921 | - |
| 2042 | 899 | 987 | - |
| 2043 | 837 | 874 | - |
| 2044 | 758 | 950 | - |
| 2045 | 612 | 724 | 106 |
